# Early Refractive and Clinical Outcomes of High-Myopic Photorefractive Keratectomy as an Alternative to LASIK Surgery in Eyes with High Preoperative Percentage of Tissue Altered

**DOI:** 10.1155/2019/6513143

**Published:** 2019-01-28

**Authors:** Nir Sorkin, Amir Rosenblatt, David Smadja, Eyal Cohen, Marcony R. Santhiago, David Varssano, Yossi Yatziv

**Affiliations:** ^1^Department of Ophthalmology, Tel Aviv Sourasky Medical Center, Tel Aviv, Israel; ^2^Department of Ophthalmology and Vision Sciences, University of Toronto, Toronto, ON, Canada; ^3^Ophthalmology Department, Shaare Zedek Medical Center, Jerusalem, Israel; ^4^Department of Ophthalmology, Federal University of Rio de Janeiro, Rio De Janeiro, Brazil

## Abstract

**Objective:**

To analyze the safety and efficacy of high-myopic PRK as an alternative to LASIK surgery in patients with a high preoperative percentage tissue altered (PTA).

**Design:**

Retrospective interventional case series.

**Participants:**

Charts of 256 consecutive eyes that underwent PRK with application of mitomycin-C 0.02% for high myopia were retrospectively reviewed.

**Methods:**

Refractive (refraction and refractive accuracy) and visual outcomes (uncorrected and corrected visual acuities), as well as occurrence of haze in the eyes with preoperative PTA expected to be higher than 40% with a 110-micron flap if undergoing LASIK surgery, were analyzed.

**Results:**

Mean follow-up was 7.3 ± 4.8 months. A total of 187 of 256 eyes (73.0%) were included in the analysis because they were expected to have a PTA greater than 40%, should they have undergone LASIK surgery. The actual mean PTA of those eyes following PRK was 31.8 ± 2.2%, and none had a PTA ≥ 40%. UDVA of 20/16, 20/20, and 20/25 or better was achieved in 1.2% (2 eyes), 65.5% (112 eyes), and 85.4% (146 eyes), respectively. The percentage of eyes with postoperative SE within ±0.5 D and ±1.0 D of planned SE was 71% and 93%, respectively. None of the eyes lost 2 or more lines of CDVA. The rate of stromal haze, managed successfully with topical steroids only, was 4.8%.

**Conclusion:**

High-myopic PRK with application of mitomycin-C in the eyes at risk of developing ectasia because of high preoperative PTA was demonstrated to be a safe and effective alternative to the LASIK procedure.

## 1. Introduction

Excimer laser refractive surgery in patients with high myopia is associated with an increased complication rate. Laser-assisted in situ keratomileusis (LASIK) in cases of high myopia carries an increased risk of iatrogenic ectasia [1]. Recently, the value of percent tissue altered (PTA) was found to be a robust indicator of the risk for post-LASIK ectasia, with PTA values over 40% indicating high ectasia risk in eyes with normal preoperative topography [2–4]. Abnormal preoperative topography is a risk factor of its own, regardless of the PTA value [1]. Photorefractive keratectomy (PRK) is considered safer than LASIK with regard to iatrogenic ectasia risk. However, PRK in high myopia has several drawbacks—it has been associated with lower efficacy, lower predictability [5–7], and a higher rate of stromal haze [6–8]. Increased rates of stromal haze in high-myopic PRK can be attributed to the deep stromal ablation performed [9]. Additionally, a smaller ablation diameter (which may be considered in large myopic corrections in order to conserve stromal tissue), is an independent risk factor for stromal haze [10].

Excimer laser technology has evolved continuously. Current generation laser platforms offer faster ablation rates, more sophisticated laser delivery methods and algorithms, optimized ablation profiles, and accurate eye tracking. Additionally, the routine application of mitomycin-C (MMC) has substantially decreased rates of stromal haze formation [11].

Nevertheless, PRK in patients with high myopia remains a challenge for refractive surgeons. Several large series (larger than 100 eyes) previously examined PRK in high myopia, using older generation laser platforms. While it was found to be safe and effective, the PRK outcome was not comparable to the outcome of PRK in low to moderate myopia [12–14]. In this study, we evaluated the safety and efficacy of the current PRK, performed with the WaveLight® EX500 excimer laser (Alcon Laboratories, Inc., Fort Worth, Texas), in a large series of patients with high myopia who would otherwise have a PTA > 40% and would be considered at high risk for ectasia if operated using LASIK.

## 2. Methods

This retrospective study was performed at the Tel Aviv Medical Center, Israel, and was conducted in accordance with the tenets of the Declaration of Helsinki. We reviewed records of patients that underwent PRK for high myopia (greater than 6D) between January 2013 and July 2013 at Care-Vision Laser Centers, Tel Aviv, Israel. The data were routinely collected and entered into the electronic medical record database by the facility staff.

### 2.1. Patients and Examination Protocol

All patients underwent a complete preoperative ophthalmological examination that included measurements of monocular uncorrected distance visual acuity (UDVA) and corrected distance visual acuity (CDVA), manifest and cycloplegic refraction, slit-lamp biomicroscopy, pupillometry, corneal topography, pachymetry, applanation tonometry, and dilated fundoscopy. Soft contact lenses and rigid gas-permeable contact lenses were removed at least 1 or 2 weeks, respectively, prior to the preoperative examination.

The eyes were included in the analysis when they met the following criteria: the eyes that underwent PRK for high myopia greater than −6 D with at least 3 months of follow-up and normal preoperative topography, defined as regular and symmetric patterns (including round, oval, or symmetric bowtie patterns) or mildly asymmetric (steepening <0.5 D and without a skewed radial axis) based on Placido disk analysis [1] and having a preoperative expected PTA greater than 40%, when the treatment was simulated with a 110-micron flap. Only eyes with the following available data were included: UDVA and CDVA, corneal topography with pachymetry and mean keratometry, and presence and grading of stromal haze according to Fantes et al. [15].

Percentage of tissue altered (PTA) has been previously described and defined for LASIK surgery as ([flap thickness + expected maximal ablation depth]/preoperative corneal thickness) × 100 [2]. PTA was defined for PRK surgery as [(expected maximal ablation depth + 50 *μ*m estimated epithelial depth)/preoperative corneal thickness] × 100.

The calculation of the mean gain in visual acuity (VA) was made on the basis of VA values obtained with Snellen decimals, and these values were converted into LogMAR. The conversion of decimal VA values according to Snellen decimal into LogMAR was made by using the following formula: LogMAR = −log(decimal VA). The final expression of VA in the decimal value was reconverted with the following formula:

Decimal VA = 10^−logMAR^. The calculation of a variation of visual acuity in terms of the number of lines was established according to the formula [(log(final decimal VA))–log(initial decimal VA)] × 10. The quantitative values were expressed as mean and standard deviation.

Reasons for choosing PRK over LASIK in this group were young age and occupational reasons (due to the mandatory military service between the ages of 18 and 21, PRK is preferred over LASIK in those patients), pachymetry less than 500 microns or predicted LASIK RSB less than 300 microns.

### 2.2. Treatment Planning and Surgical Technique

All the PRK procedures were performed by 6 different ophthalmic surgeons according to the same surgical protocol and with the wavefront-optimized photoablation profile (WFO) using the WaveLight EX500 excimer laser (Alcon Laboratories, Inc., Fort Worth, Texas). The surgical procedures were performed with a 0.95 mm spot size, an optical zone of either 6.00 or 6.50 mm, and a transition zone of 1.25 mm. The magnitude of the spherical and astigmatic correction was first determined using the WaveLight EX500 platform's integrated nomogram, and adjustments were made by the surgeon based on the final assessment of all available clinical data.

Epithelium removal was performed using 20% ethanol placed on the cornea in an 8.5 mm well for 15 seconds. The cornea was then rinsed with a balanced salt solution, and a blunt spatula was used to peel off the epithelium followed by stromal ablation using the excimer EX500 excimer laser. In all the eyes, a sponge soaked with 0.02% MMC was placed on the stroma for 30 to 90 seconds (according to the depth of the ablation) immediately after excimer ablation. After rinsing to remove the MMC, a contact lens was placed. Following surgery, moxifloxacin 0.5% 4 times daily for 1 week, dexamethasone 0.1% 4 times daily for 4 weeks followed by a taper, and preservative-free artificial tears for several months were prescribed. The eyes were examined for 1 day, 1 and 6 weeks, and 3 and 12 months postoperatively. UDVA, CDVA manifest refraction, and slit-lamp biomicroscopy were evaluated at every postoperative examination. Corneal topography was performed at 6 weeks, 3 months, and 12 months postoperatively.

### 2.3. Data Analysis and Statistics

Data were recorded in Microsoft Excel and analyzed using SPSS version 21 (SPSS Inc., Chicago, IL, USA). Postoperative data were collected at the 3rd month and at the last follow-up visit for the analysis. Continuous variables, such as visual acuity, were compared within subjects using the paired *t*-test and between subjects using the independent sample *t*-test. In cases of multiple independent comparisons, one way ANOVA was used.

For small group comparison and ordinal variables, the Wilcoxon nonparametric test was used for paired comparisons and the Mann–Whitney nonparametric test was used for independent samples. Multiple independent comparisons were performed by the Kruskal-Wallis nonparametric test.

Binary variables were compared within subjects using the McNemar test for symmetry and between subjects using Fisher's exact test or Pearson chi-square test. To analyze the correlation between continuous variables, Pearson's correlation test was used. Univariate analysis and multivariate logistic regression models were constructed in order to test the association between multiple relevant covariates and haze formation. All tests were 2-tailed, and the threshold for statistical significance was defined as a *p* value <0.05.

## 3. Results

This study included 256 highly myopic eyes of 129 subjects, and 106 eyes were eyes of female participants (41.4%). The mean follow-up time was 7.3 ± 4.8 months. Baseline clinical and demographic characteristics of the subjects are summarized in Table 1.

### 3.1. Eyes with Simulated LASIK PTA Larger than 40%

Of the 256 highly myopic eyes that underwent PRK, none had actual PRK PTA >40%. The average PTA was 30.5 ± 3.0% (range 22.9% to 38.0%). If those eyes would have had LASIK performed, 187 eyes (73.0%) would have had a PTA > 40% with a 110-micron flap and 134 eyes (52.3%) would have had a PTA > 40% with a 100-micron flap.  Table 2 compares the demographic and preoperative data of these groups.

Efficacy and safety analysis was performed for the patients with expected PTA > 40% in 110-micron flap LASIK. All 187 analyzed eyes had at least 3 months of follow-up, and 65 of 187 eyes were examined at the 12-month follow-up. Outcomes of the 3rd month and of the last available follow-up visit are presented (last follow-up time ranges from 3 to 20 months).

### 3.2. Predictability

**Figure 1:**
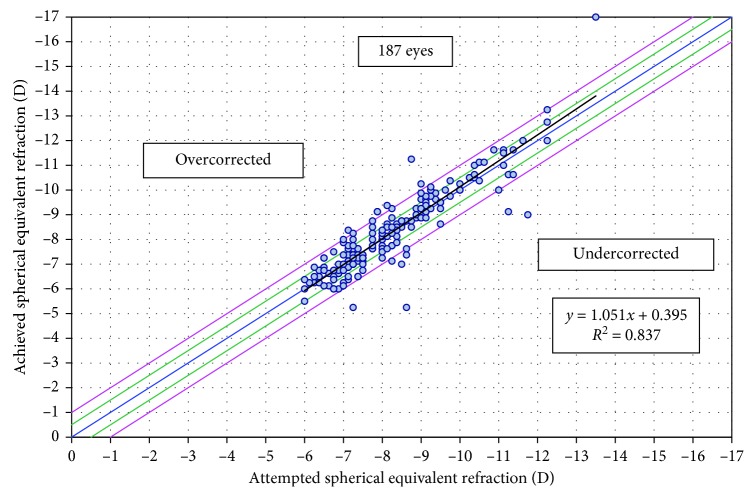
Comparison of achieved versus attempted spherical equivalents.

Postoperative SE at 3 months and at the final follow-up was within ±0.5 D of the target SE in 70.4% and 71.0% of the eyes, respectively, and within ±1.00 D in 92.2% and 93.0% of the eyes, respectively. Refractive astigmatism at 3 months and at the final follow-up was within ±0.50 D in 77.1% and 72.0% of the eyes, respectively, and within ±1.00 D in 96.7% and 90.0% of the eyes, respectively.  Figure 1 shows the correlation between attempted and achieved SE at the final follow-up. Six eyes of 5 patients (3.2%) underwent an enhancement procedure at 31.7 ± 29.2 months (range 12.0 to 69.4 months). There were no enhancement procedures performed during the first postoperative year.

### 3.3. Efficacy

Of the 171 eyes corrected for distance, UCVA of 20/16, 20/20, 20/25, 20/40, or better at 3 months was achieved in 0 eyes (0.0%), 57 eyes (33.3%), 140 eyes (81.9%), and 168 eyes (98.2%), respectively. UCVA of 20/16, 20/20, 20/25, 20/40, or better at the final follow-up was achieved in 2 eyes (1.2%), 112 eyes (65.5%), 146 eyes (85.4%), and 170 eyes (99.4%), respectively. The efficacy index was 1.017.

### 3.4. Safety

Postoperative CDVA at 3 months (0.89, range: 0.40 to 1.20) and at the final follow-up (0.88, range: 0.45 to 1.00) was significantly better (*p* < 0.001 for both) than preoperative CDVA (0.85, range 0.40 to 1.00).

One eye (0.5%) gained 2 lines of CDVA. There were no eyes that lost 2 or more lines of CDVA. The safety index was 1.046.

### 3.5. Complications

All procedures were uneventful. There were no major postoperative complications. Stromal haze was documented in 9 eyes of 6 patients (4.8% of 187 eyes). The degree of haze was grade 1 in 8/9 eyes and grade 3 in 1/9 eyes. Management in all cases consisted of a change in the topical steroidal regimen. None of the eyes required further intervention due to haze. There were no cases of corneal ectasia.

### 3.6. Difference between Simulated PTA Groups

There were no significant final follow-up outcome differences between the simulated PTA > 40% groups and the entire PRK cohort (Table 3).

## 4. Discussion

In this study, we evaluated the efficacy and safety of PRK, performed with a new-generation excimer laser platform, in a large series of eyes with high myopia which would otherwise have a PTA > 40% and would be considered at high risk for ectasia if operated using LASIK.

A total of 65.5% of the eyes corrected for distance had UCVA of 20/20 or better, and 99.4% had UCVA of 20/40 or better. The postoperative SE was within ±0.5 D of the target SE in 71% of the eyes and within ±1.00 D in 93% of the eyes. Several large series (more than 100 eyes in each) investigated PRK in high myopia [12–14]. Their results are shown in Table 4. The efficacy of PRK in our highly myopic cohort was superior to that of similar older studies. This can be attributed to technological advancements in excimer laser technology and the current routine application of MMC.

We were unable to find large cohorts of highly myopic eyes who underwent PRK in the recent literature. A recent study evaluated 77 eyes with high myopia that underwent either PRK with MMC or LASEK, both using the LADARVision 4000 excimer laser system (Alcon Surgical, Inc., Ft. Worth, TX). In this study, 84.4% of the eyes had postoperative UCVA of 20/20 or better [16]. This may appear more favorable than our outcome (65.5% in our study with postoperative UCVA of 20/20 or better), but in our study, only 58.2% of the eyes had preoperative CDVA of 20/20 or better, compared with 100% in that study [16].

In a recent study, femto-LASIK performed on 134 highly myopic eyes showed a 3-month predictability of 60.4% and 87.3% for SE within ±0.50 D and ±1.00 D, respectively. UCVA of 20/20 or better was documented in 82.8% of the patients, while preoperative CDVA of 20/20 or better was documented in 89.6% of the patients [17]. Wavefront-guided LASIK using a new-generation Hartmann–Shack aberrometer performed on a series of 621 eyes showed a slightly improved outcome with predictability indices of 82.6% and 95.0% for SE within ±0.50 D and ±1.00 D, respectively. UCVA of 20/20 or better was documented in 82.4% of the patients [18]. A similar efficacy profile was seen with wavefront-optimized WaveLight EX500 ablation in femto-LASIK performed on 160 highly myopic eyes [19]. Thus, it seems that our cohort of highly myopic PRK showed predictability comparable to some of the data on current high-myopic LASIK. Postoperative UCVA data appear more favorable in LASIK [17–19]. However, in those studies, preoperative CDVA was much better than the preoperative CDVA of our cohort.

While efficacy of LASIK may be slightly superior to that of PRK in patients with high myopia, the risk of ectasia may be substantially increased due to the deep stromal alteration. The value of PTA was found to be a robust indicator of the risk for post-LASIK ectasia, and a value of more than 40% is associated with increased ectasia risk [2]. The average actual PTA of our PRK patients was 30.5 ± 3.0% with none of them having a PTA > 40% (range 22.9% to 38.0%). If those eyes would have had LASIK performed using a 110-micron flap, 187 eyes (73.0%) would have had a PTA > 40% with a 110-micron flap. The subgroup of patients with simulated LASIK PTA > 40% showed efficacy similar to that of our entire highly myopic PRK cohort. The safety profile in our study was good. There were no cases of corneal ectasia, and no eyes lost 2 or more lines of CDVA. PRK in high myopia has previously been associated with an increased risk for the development of stromal haze [6–8]. Stromal haze in this study was seen in 4.8% and was clinically insignificant in all cases but one. This rate of stromal haze is substantially lower than that of similar studies of high-myopic PRK [12–14] that were published prior to the MMC era. Smaller series of high-myopic MMC-PRK in the recent literature showed the percentage of haze formation to be between 3% and 12.2% [16, 20, 21]. Therefore, it can be concluded that haze rates in high-myopic PRK today are much lower than previously reported, probably due to routine MMC use, and that haze formation is in the large part clinically inconsequential.

This study was aimed at showing early outcomes of high-myopic PRK as an alternative to LASIK in high-risk eyes. The risk for ectasia in high-myopic PRK patients should be evaluated in the long-term, although ectasia following PRK is extremely rare and has been recently shown to be 0.029% (9 of 31,045 eyes), with none of the ectasia cases being highly myopic [22]. Further long-term studies can also evaluate the rate of regression of high-myopic PRK in the MMC era.

In conclusion, current PRK in the eyes with high myopia is efficacious, accurate, and safe. In cases with high ectasia risk due to increased PTA, PRK is a solid alternative to LASIK.

## Figures and Tables

**Table 1:** Baseline clinical and demographic characteristics.

	Mean ± SD	Range
Age (years)	25.8 ± 7.1	16 to 55
Sphere (D)	−7.36 ± 1.59	−4.50 to −13.50
Cylinder (D)	−0.92 ± 0.80	0.00 to −4.50
Spherical equivalent (D)	−7.82 ± 1.62	−6.00 to −14.00
Preoperative CDVA (Snellen decimals)	0.86	0.45 to 1.00
Average keratometry (D)	44.5 ± 1.5	40.7 to 49.9
Pachymetry (*µ*m)	524.5 ± 31.1	451 to 633

SD = standard deviation; D = diopters; CDVA = corrected distance visual acuity.

**Table 2:** Patients with expected PTA > 40% for 110-micron LASIK and 100-micron LASIK.

	110-micron LASIK (*n*=187) (mean ± SD)	100-micron LASIK (*n*=134) (mean ± SD)	*p* value
Simulated average PTA (%)	43.5 ± 2.2	42.6 ± 1.9	0.001
Age (years)	25.6 ± 6.4	25.7 ± 6.7	0.943
Spherical equivalent (D)	−8.20 ± 1.68	−8.62 ± 1.71	0.029
Average keratometry (D)	44.6 ± 1.5	44.7 ± 1.6	0.745
Pachymetry (*µ*m)	515.1 ± 27.3	512.9 ± 27.5	0.473
Preoperative CDVA (Snellen decimals)	0.85	0.83	0.364

SD = standard deviation; D = diopters; PTA = percent tissue altered; CDVA = corrected distance visual acuity; UDVA = uncorrected distance visual acuity.

**Table 3:** Comparison of results between the entire highly myopic PRK cohort and subgroups of PRK patients that would have had a PTA >40% if they had undergone either 110-micron LASIK or 100-micron LASIK.

	PTA > 40% with 110-micron LASIK (*n*=187)	PTA > 40% with 100-micron LASIK (*n*=134)	Entire PRK cohort *n*=256	*p* value
Actual average PTA with PRK (%) (mean ± SD)	31.8 ± 2.2	32.8 ± 1.9	30.5 ± 3.0	<0.001
Final SE within ± 0.50 D (*n* (%))	132 (71%)	91 (68%)	183 (71%)	0.921
Final SE within ± 1.00 D (*n* (%))	173 (93%)	122 (91%)	240 (94%)	0.914
Postoperative CDVA (Snellen decimals)	0.88	0.87	0.89	0.221
Postoperative UDVA (Snellen decimals)	0.84	0.83	0.85	0.713
Safety index	1.046	1.050	1.050	0.945
Efficacy index	1.017	1.018	1.016	0.992
Stromal haze (*n* (%))	9 (4.8%)	6 (4.5%)	10 (3.9%)	0.894

SD = standard deviation; D = diopters; PTA = percent tissue altered; CDVA = corrected distance visual acuity; UDVA = uncorrected distance visual acuity.

**Table 4:** Large published series of photorefractive keratectomy in high myopia.

Series	Number of eyes	Surgery dates	Excimer laser platform	UCVA (%)		Spherical equivalent error		CDVA line change		Haze	MMC used
				>20/20	>20/40	±0.50 D	±1.00 D	Gain ≥1	Loss ≥2		
Our series	187	2013	WaveLight EX500	65.5%	99.4%	71.0%	93.0%	7.4%	0.0%	4.8%	Yes
Alio et al. [12]^*∗*^	267	1992–5	VISX 20/20	10.0%	63.0%	40.0%	58.0%	52%	11.6%	8.6%	No
Cennamo et al. [13]	452	1991–8	Aesculap	—	49.0%	—	49.8%	—	15.7%	∼70%	No
Steinert and Hersh [14]	210	<1998	Summit Apex	29–35%	71–74%	22–40%	47–68%	—	—	—	No

^*∗*^10-year follow-up. UCVA = uncorrected visual acuity; CDVA = corrected distance visual acuity; D = diopters; MMC = mitomycin-C. The WaveLight EX500 is manufactured by Alcon Laboratories, Inc., Fort Worth, TX, USA. The VISX 20/20 is manufactured by VISX, Inc., Santa Clara, CA, USA. The Aesculap is manufactured by Aesculap-Meditec, Jena, Germany. The Summit Apex is manufactured by Summit Technology, Inc., Waltham, MA, USA.

## Data Availability

The corresponding author had full access to all the data in the study and takes responsibility for the integrity of the data and the accuracy of the data analysis as well as the decision to submit for publication.
